# IgE by Itself Affects Mature Rat Mast Cell Preformed and *De Novo*-Synthesized Mediator Release and Amplifies Mast Cell Migratory Response

**DOI:** 10.1371/journal.pone.0079286

**Published:** 2013-10-30

**Authors:** Aleksandra Słodka, Magdalena Wiktorska, Ewa Brzezińska-Błaszczyk

**Affiliations:** 1 Department of Experimental Immunology, Medical University of Łódź, Łódź, Poland; 2 Department of Molecular and Medical Biophysics, Medical University of Łódź, Łódź, Poland; Federal University of São Paulo, Brazil

## Abstract

**Background:**

Immunoglobulin E (IgE) binds to high affinity receptor FcεRI numerously expressed on mast cells. Recent findings have revealed that IgE by itself may regulate various aspects of mast cell biology, however, detailed data is still limited.

**Methodology/Findings:**

Here, we have examined the influence of IgE alone, used at different concentrations, on mast cell activity and releasability. For the study we have employed *in vivo* differentiated mature tissue mast cells isolated from rat peritoneal cavity. Mast cells were exposed to IgE alone and then the release of preformed and *de novo*-synthesized mediators, surface FcεRI expression and mast cell migratory response were assessed. IgE by itself was found to up-regulate FcεRI expression and activate mast cells to degranulation, as well as *de novo* synthesis and release of cysteinyl leukotrienes and TNF. We have provided evidence that IgE alone also amplified spontaneous and CCL5- or TNF-induced migration of mast cells. Importantly, IgE was effective only at concentrations ≥ 3 µg/mL. A molecular basis investigation using an array of specific inhibitors showed that Src kinases, PLC/PLA_2_, MAP kinases (ERK and p38) and PI3K were entirely or partially involved in IgE-induced mast cell response. Furthermore, IgE alone stimulated the phosphorylation of MAP kinases and PI3K in rat mast cells.

**Conclusion:**

Our results clearly demonstrated that IgE by itself, at higher concentrations, influences mast cell activity and releasability. As there are different conditions when the IgE level is raised it might be supposed that *in vivo* IgE is one of the important factors modulating mast cell biology within tissues.

## Introduction

Under physiological conditions, immunoglobulin E (IgE) synthesis and, hence its concentration in the blood or within tissues is constantly low [1]. In certain disorders, however, overproduction of IgE occurs. It is well known that in the course of allergic diseases and during the host response to parasite infection, IgE synthesis rises dramatically [2], [3]. An elevated level of IgE is observed in some primary immunodeficiencies such as hyper-IgE syndrome (Job's syndrome) [4], [5], Wiskott-Aldrich syndrome and DiGeorge syndrome [6]. High serum IgE concentration is also detected in some lymphoproliferative malignancies [7]–[10]. Increased serum IgE level has been noticed in HIV-1 infection [11], [12] and this elevation was not a reflection of higher prevalence of atopic diseases among infected subjects [13]. Some data suggests that in Kawasaki disease [14] and in the course of psoriasis [15] IgE level is raised. Interestingly, specific IgE autoantibodies are detected during certain autoimmune diseases such as rheumatoid arthritis [16], [17] and bullous pemphigoid [18].

IgE binds with high affinity to the FcεRI abundantly expressed on mast cells. Mast cells are widely distributed throughout the body and are the source of large numbers of biologically active mediators. Mast cell-derived mediators exert diverse proinflammatory, anti-inflammatory, and/or immunoregulatory effects and modulate the activity of many cell populations. Thus, mast cells participate not only in maintaining homeostasis mainly *via* their involvement in angiogenesis, tissue remodeling and repair, but also are key players in inflammatory processes and the host response to pathogens. Moreover, mast cells play an important role in the course of many diseases, other than IgE-dependent hypersensitivity reactions, for example, atherosclerosis, rheumatoid arthritis, congestive heart failure, malignancies, Crohn's disease and pulmonary fibrosis [1], [19]–[21]. Mast cell activities within tissues can be regulated by different immunological and non-immunological factors such as various cytokines/chemokines, products of complement activation, bacterial and viral components, neuropeptides or IgGs [19], [20]. Interestingly, it has been indicated that IgE by itself (so called monomeric IgE), without a cross-linking agent, also influences various aspects of mast cell biology and activity. IgE binding to FcεRI causes dose- and time-dependent up-regulation of surface FcεRI expression on different mast cell lines, including mouse bone marrow-derived mast cells (BMMCs), cord blood-derived mast cells (CBMCs) and mice peritoneal mast cells [22]–[25]. IgE alone promotes prolonged cell survival, likely by preventing mast cell apoptosis [22], [26]–[29], and can initiate mast cell adhesion to the extracellular matrix (ECM) component fibronectin as well [30]. Kitaura et al. [31] indicated that the IgE molecule may act as a mast cell chemoattractant. Some studies have suggested that IgE by itself triggers mast cell to generate and release various mediators [26], [28], [32]–[34]. Tanaka et al. [32], [35] clearly demonstrated that IgE alone was able to up-regulate histidine decarboxylase activity, leading to histamine synthesis in BMMCs, and that this process was highly dependent on transient mobilization of cytosolic Ca^2+^. Of note, the majority of studies were carried out using cell lines differentiated *in vitro* (e.g. BMMCs, CBMCs), which differ with respect to phenotype and activity from mast cells, which develop and mature under influence of microenvironmental factors. It should be also emphasized that the available data concerning the influence of IgE exposure on mast cell response and activity still remains limited.

In the present study we scheduled to examine the direct impact of IgE alone, used at different concentrations, on various effector functions of fully mature rat peritoneal connective tissue mast cells. To this end, we examined the IgE-induced mast cell preformed mediators release, arachidonic acid metabolite generation and tumor necrosis factor (TNF) *de novo* synthesis. Another important question to clarify was whether IgE alone affects spontaneous migratory response of mast cells, as well as migration induced by strong mast cell chemotactic factors such as CCL5 and TNF [36]–[38]. FcεRI expression on native and IgE-coated mast cells was also examined. To better understand the molecular basis of IgE-induced mast cell responses, we investigated the participation of some signaling molecules involved in mast cell activation *via* FcεRI, i.e. mitogen-activated protein (MAP) kinases (extracellular signal-regulated kinase (ERK) and p38 kinase), Src kinases, phosphatidylinositol 3-kinase (PI3K) and phospholipase (PL)C/PLA_2_.

## Materials and Methods

### Reagents

Dulbecco's Modified Eagle's Medium (DMEM), Hank's balanced salt solution (HBSS), sodium bicarbonate, fetal calf serum (FCS), gentamicin and glutamine were purchased from GIBCO, Life Technologies (Gaithersburg, MD, USA). NaCl, KCl, MgCl_2_, CaCl_2_, 2-hydroxyethylpiperazine-N'-ethanesulphonic acid (HEPES), NaOH, glucose, HCl, *o*-phthaldehyde (OPT), Percoll, hematoxylin, toluidine blue, trypan blue, bovine serum albumin (BSA), PLC/PLA_2_ inhibitor (U73122), PI3K inhibitor (LY294002), Src kinase inhibitor (Src Inhibitor-1, Src I-1), RIPA buffer and laminin from human placenta were obtained from Sigma-Aldrich (St. Louis, MO, USA). ERK inhibitor (PD98059) and p38 kinase inhibitor (SB203580) were purchased from InvivoGen (San Diego, CA, USA). Recombinant rat (rr) tumor necrosis factor (TNF) was obtained from R&D Systems (Minneapolis, MN, USA), rrCCL5, and mouse anti-rat IgE monoclonal IgG1 antibodies (anti-IgE) were purchased from AbD Serotec (Oxford, UK). Mouse monoclonal IgG1 anti-FcεRI and anti-PI3K antibodies were obtained from Abcam Inc. (Cambridge, MA, USA) and purified rat myeloma IgE was obtained from Invitrogen, Life Technologies (Gaithersburg, MD, USA). Fluorescein isothiocyanate (FITC)-labeled goat anti-mouse polyclonal antibodies, purified mouse monoclonal IgG1 antibodies (isotype control), and BD CellFIX were purchased from BD Biosciences (Benelux, NV, Belgium). The 48-well Boyden microchamber as well as the 8-μm-pore-size polycarbonate filters were purchased from Neuro Probe (Gaithersburg, MD, USA). Rat TNF and cysLT specific immunoassay kits were purchased from Gen-Probe Inc. (San Diego, CA, USA) and Cayman Chemical (Ann Arbor, MI, USA), respectively. The RNeasy Mini Kit was obtained from Qiagen (Hilden, Germany). The High Capacity cDNA Reverse Transcription Kit, TaqMan® probes dyed 6-carboxyfluorescein (6-FAM) (rat TNF Rn01525859 and rat Actb Rn00667869) and TaqMan® Gene Expression Master Mix were purchased from Applied Biosystems (Foster City, CA, USA) and bicinchoninic acid (BCA) Protein Assay Kit was obtained from Pierce (Rockford, IL, USA). The Western Lightning luminol-based enhanced chemiluminescence (ECL) Pro system was purchased from Perkin-Elmer (Boston, MA, USA). Anti-phospho-ERK1/2 (Thr-202/Tyr-204) and anti-ERK1/2, anti-phospho-p38 (Tyr-182) and anti-p38 as well as horseradish peroxidase (HRP)-labeled donkey anti-goat IgG, goat anti-mouse IgG-HRP and goat anti-rabbit IgG-HRP all were obtained from Santa Cruz Biotechnology Inc. (Santa Cruz, CA, USA). Anti-phospho-PI3K (p85 Tyr458/p55 Tyr199) antibodies were obtained from Thermo Scientific (Rockford, IL, USA).

### Experimental Animals

Mast cells were collected from peritoneal cavities of female Wistar rats weighing ∼250 g, aged 3–4 months, bred in the animal quarters of the Medical Faculty of the Medical University of Łódź. Standard storage conditions for animals were provided, i.e. room temperature (20±2°C), artificial lighting for 12 h and 12 h of darkness, in metal cages, 5 rats in each. The animals were fed with LSM Murigran granulated fodder for rodents and water *ad libitum*. Isoflurane-induced anesthesia was carried out prior to animal decapitation. All efforts were made to minimize animal suffering. Animal experiments were approved by the Local Ethics Committee for Experiments on Animals of the Medical University of Łódź (approval number: 42/ŁB 529/2010). All manipulations performed on animals were in accordance with university guidelines.

### Isolation of Mast Cells

Peritoneal cell suspensions were obtained from peritoneal cavities by lavage with 50 mL of 1% HBSS supplemented with 0.015% sodium bicarbonate. Peritoneal cell suspension was washed twice (1200 rpm, 5 min, 20°C) in complete (c)DMEM containing DMEM supplemented with 10% FCS, 10 µg/mL gentamicin, and 2 mM glutamine. In order to collect purified mast cells, peritoneal cells were resuspended in 72.5% isotonic Percoll solution and centrifuged (1500 rpm, 20 min, 20°C). After being washed twice (1200 rpm, 5 min, 20°C), mast cells were counted and resuspended in an appropriate volume of cDMEM (for TNF synthesis determination, migration assay and flow cytometry analysis) or in medium for rat mast cells, containing 137 mM NaCl, 2.7 mM KCl, 1 mM MgCl_2_, 1 mM CaCl_2_, 10 mM HEPES, 5.6 mM glucose, and 1 mg/mL BSA (for histamine release assay and cysLT synthesis measurement), to obtain mast cell concentration of 1.5×10^6^ cells/mL. Mast cell were prepared with purity > 98%, as determined by toluidine blue staining.

### Histamine Release Assay

Purified mast cells were incubated with IgE at final concentrations of 0.1, 0.5, 1, 1.5, 3, or 5 µg/mL, or without IgE (negative control) in a water bath for 1 h at 37°C with constant stirring. In every experiment a positive control for the determination of anti-IgE-induced histamine release from native mast cells (final concentration of anti-IgE - 5 µg/mL, time of incubation – 1 h) was included. For time-course experiments mast cells were incubated with IgE at final concentrations of 5 µg/mL for 0, 5, 10, 30, or 60 min. After incubation the reaction was stopped by adding 1.9 mL of cold medium for rat mast cells and the release of histamine was measured using spectrofluorometric method, as described previously [39]. Briefly, after centrifugation (1200 rpm, 5 min, 4°C), supernatants were decanted into separate tubes whereas 2 mL of distilled water was added to each tube with cell pellet. All samples were acidified with 3 N HCL. The histamine content was determined in both cell pellets (residual histamine) and supernatants (released histamine) using OPT. Histamine release was expressed as a percentage of the total cellular content of this amine.

### CysLT Synthesis Measurement

Purified mast cells were incubated with IgE at final concentrations of 0.1, 0.5, 1, 1.5, 3, or 5 µg/mL, or without IgE (negative control) in a water bath for 1 h at 37°C with constant stirring. In every experiment a positive control for the determination of native mast cell cysLT synthesis in response to anti-IgE stimulation (final concentration of anti-IgE - 5 µg/mL, time of incubation - 1 h) was included. The supernatants were collected by centrifugation (1200 rpm, 5 min, 20°C) and analyzed for cysLT by an ELISA commercial kit according to the manufacturer's instructions. The sensitivity of this assay was < 13 pg/mL.

### TNF Generation Analysis

Purified mast cells were incubated with IgE at final concentrations of 0.1, 0.5, 1, 1.5, 3, or 5 µg/mL, or without IgE (negative control) for 6 h at 37°C in a humidified atmosphere with CO_2_. Native mast cells were treated with anti-IgE at concentration of 5 µg/mL under the same experimental conditions (positive control). The supernatants were collected by centrifugation (1200 rpm, 5 min, 20°C) and analyzed for TNF by an ELISA commercial kit according to the manufacturer's instructions. The sensitivity of this assay was < 20 pg/mL.

### Quantitive RT-PCR

Purified peritoneal rat mast cells were stimulated with IgE or anti-IgE (positive control) both at 5 µg/mL for 0, 15, 30, 60, 180, and 360 min at 37°C in a humidified atmosphere with 5% CO_2_. Total RNA was isolated from mast cells using an RNeasy Mini Kit. Synthesis of cDNA was performed using High Capacity cDNA Reverse Transcription Kit. For qRT-PCR, TaqMan® probes and TaqMan® Gene Expression Master Mix were used. All reactions were performed with the use of 7900 HT Fast Real-Time PCR System (Applied Biosystems). The expression of TNF mRNA was corrected by normalization based on the transcript level of the housekeeping gene rat Actb. All qRT-PCRs were conducted in triplicate, and the changes in gene expression are presented as fold-increases relative to non-stimulated mast cells.

### Migration Assay

Mast cell migration was analyzed in a 48-well Boyden microchamber using 8-μm-pore-size polycarbonate filters. The filters were coated overnight at room temperature with laminin at a concentration of 100 µg/mL and air dried for at least 1 h before use. Purified mast cells were preincubated with IgE at 1 µg/mL and 5 µg/mL or medium alone (native mast cells) for 1 h at 37°C with constant stirring. After preincubation, mast cells were washed in cDMEM (1200 rpm, 5 min, 20°C). Next, 30 µL of rrTNF at 10 ng/mL, rrCCL5 at 100 ng/mL or medium alone (spontaneous migration) was placed into the lower compartment of a microchamber, and 50 µL of native or IgE-coated mast cells was pipetted into the upper compartments. The mast cells were incubated in the chemotaxis chamber for 3 h at 37°C in a humidified incubator with 5% CO_2_. After incubation, the non-migrating cells were removed from the upper surface of the filter by scraping, and migrating cells adherent to the lower surface of the membrane were fixed in 99.8% ethanol, washed in distilled water, stained for 10 min with hematoxylin, cleared in distilled water, and mounted on microscope slides. Mast cell migration was quantified by counting the total number of cells migrating through the filter in 10 high power fields (HPF).

### Mast Cell Treatment with Signaling Transduction Pathway Inhibitors

In independent experiments, mast cells were pretreated with various signaling pathway inhibitors for 1 h at 37°C in a water bath with constant stirring, before main procedure accomplishments (i.e. histamine release assay, cysLT synthesis measurement, TNF generation analysis, or migration assay). Src kinase inhibitor (Src I-1) was used at a concentration of 0.01 µM, PLC/PLA_2_ inhibitor (U73122) was used at a concentration of 0.1 µM, ERK kinase inhibitor (PD98059) was used at a concentration of 100 µM, PI3K inhibitor (LY294002) was used at a concentration of 50 µM, and p38 inhibitor (SB203580) was used at a concentration of 100 µM. It should be underlined that the concentrations of all applied inhibitors were chosen in the preliminary experiments, and neither of inhibitors affected mast cell viability, as examined by staining with trypan blue.

### Western Blotting

After incubation with 5 µg/mL of IgE alone for either 0, 5, 15, 30, 60 or 120 min, purified mast cells were washed with PBS and lysed in RIPA buffer on ice for 20 min, sonicated and centrifuged (14 000 rpm, 5 min, 20°C). Total protein of the cell lysate was assessed by BCA reagent. Cell lysates containing 50 µg of crude cell lysate protein were analyzed by 10% SDS-PAGE followed by immunoblotting. Protein expression was detected using anti-phospho-ERK1/2 (Thr-202/Tyr-204), anti-phospho-p38 (Tyr-182) or anti-phospho-PI3K (p85 Tyr458/p55 Tyr199) antibodies, and appropriate antibodies that recognize these MAP kinases or PI3K, irrespective of their phosphorylation states. HRP-conjugated anti-mouse, anti-goat or anti-rabbit IgGs were used as secondary antibodies and then visualized using chemiluminescence reagents.

### Flow Cytometry

Purified mast cells, at a concentration of 2×10^6^ cells/mL, were incubated with IgE at 1 µg/mL or 5 µg/mL or without IgE (native mast cells) for 1, 6 or 24 h at 37°C in a humidified atmosphere with 5% CO_2_. After incubation, cells were washed with cDMEM in order to remove unbound IgE, fixed using 1% CellFIX solution for 15 min at 4°C, washed twice, and finally resuspended in 1% PBS. For the measurement of FcεRI expression, mast cells were initially incubated with mouse anti-rat FcεRI IgG1 antibodies or mouse IgG1 isotype control with irrelevant specificity (both at a final concentration of 10 µg/mL) at 4°C for 45 min. After washing with 1% PBS, cells were stained with FITC-conjugated goat anti-mouse IgG1 antibodies at a final concentration of 5 µg/mL at 4°C for 45 min in the dark. Following this, the cells were washed twice and finally resuspended in 300 µL of 1% PBS prior to fluorescence assessment. Ten thousand events in each sample were analyzed using a FACSCalibur flow cytometer with CellQuest software (BD Biosciences). IgE-dependent mast cell FcεRI expression was presented as a percentage of FcεRI MFI (mean fluorescence intensity) measured in native mast cells (referred to as 100%). After each period of incubation mast cell viability was examined using the trypan blue exclusion test.

### Statistical Analysis

Results are expressed as mean value ± standard error of the mean (SEM) and tested statistically by Student's t-test for “small groups”. Results were considered statistically significant when p<0.05.

## Results

### IgE Alone Induces Mast Cell Degranulation

**Figure 1:**
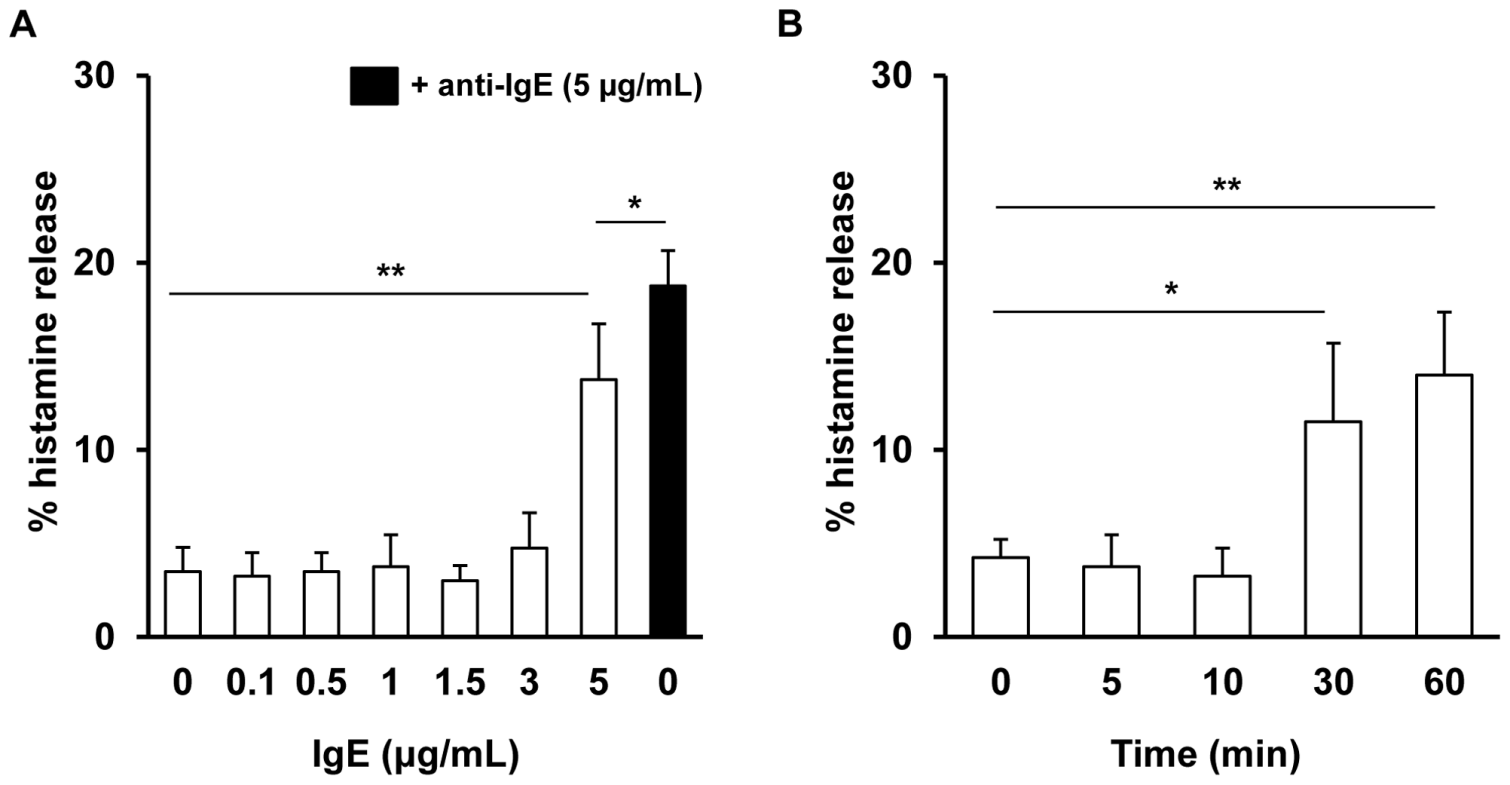
IgE alone induces mast cell degranulation. Mast cells were incubated with different concentrations of IgE (white bars) for 1 h. Anti-IgE-induced histamine release (black bar) is shown as a positive control (A). Mast cells were stimulated with IgE at 5 µg/mL for indicated times of incubation (B). Results are presented as the mean ± SEM of at least four independent experiments and each experiment was carried out in duplicate (n≥4). *p<0.05, **p<0.01.

The effects of various concentrations of IgE, from 0.1 µg/mL to 5 µg/mL, on mast cell degranulation and preformed mediator release were evaluated first. We found that IgE alone, used at concentrations of 0.1, 0.5, 1, 1.5, and 3 µg/mL, failed to induce detectable mast cell degranulation and histamine release. However, it was noted that when stimulated with 5 µg/mL of IgE for 1 h, mast cells were activated to secrete histamine. The level of 5 µg/mL IgE-induced histamine release was significantly (p<0.01) higher than in control but statistically (p<0.05) lower than anti-IgE-challenged histamine secretion (Fig. 1A). Time-course studies revealed that 30 min stimulation resulted in statistically significant IgE-induced histamine release as compared to control (Fig. 1B).

### IgE Alone Stimulates Mast Cells to CysLT and TNF Production

**Figure 2:**
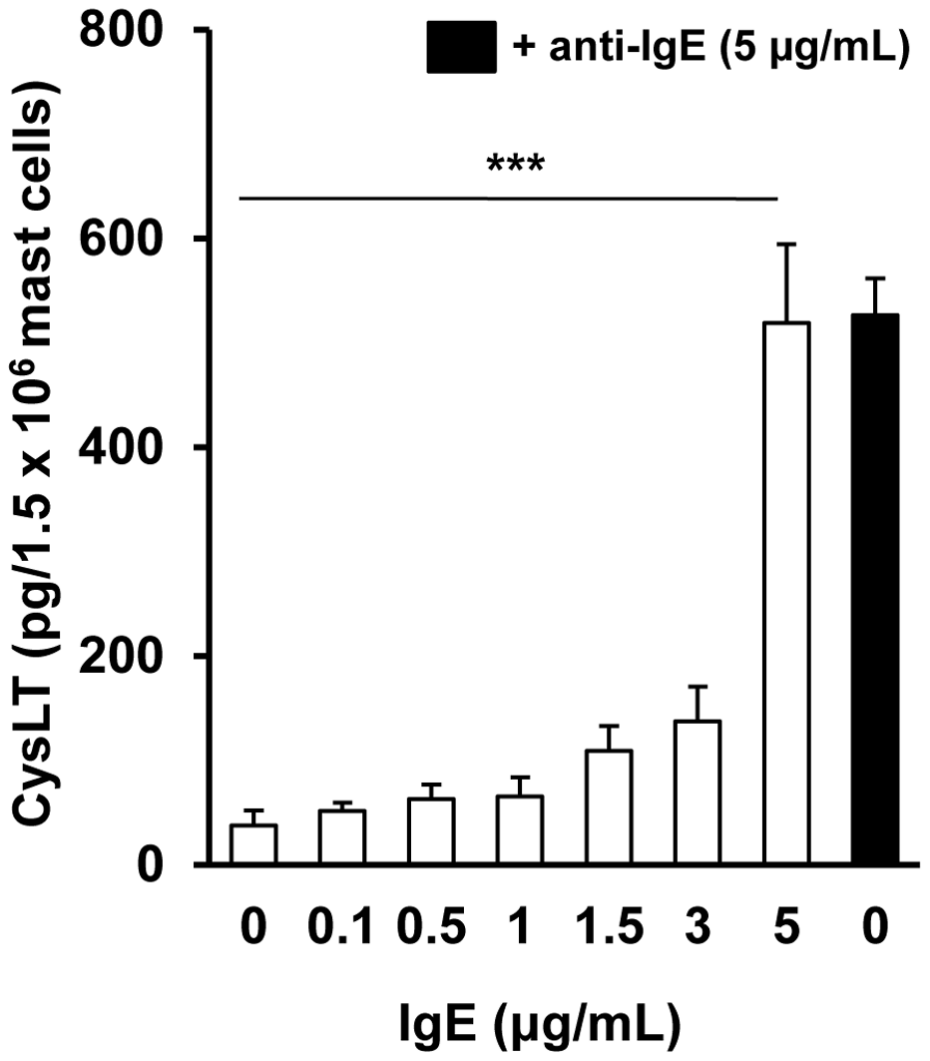
IgE alone stimulates mast cells to cysLT synthesis. Mast cells were incubated with different concentrations of IgE (white bars) for 1 h. Anti-IgE-induced cysLT secretion (black bar) is presented as a positive control. Results are shown as the mean ± SEM of four independent experiments and each experiment was carried out in duplicate (n = 4). ***p<0.001.

**Figure 3:**
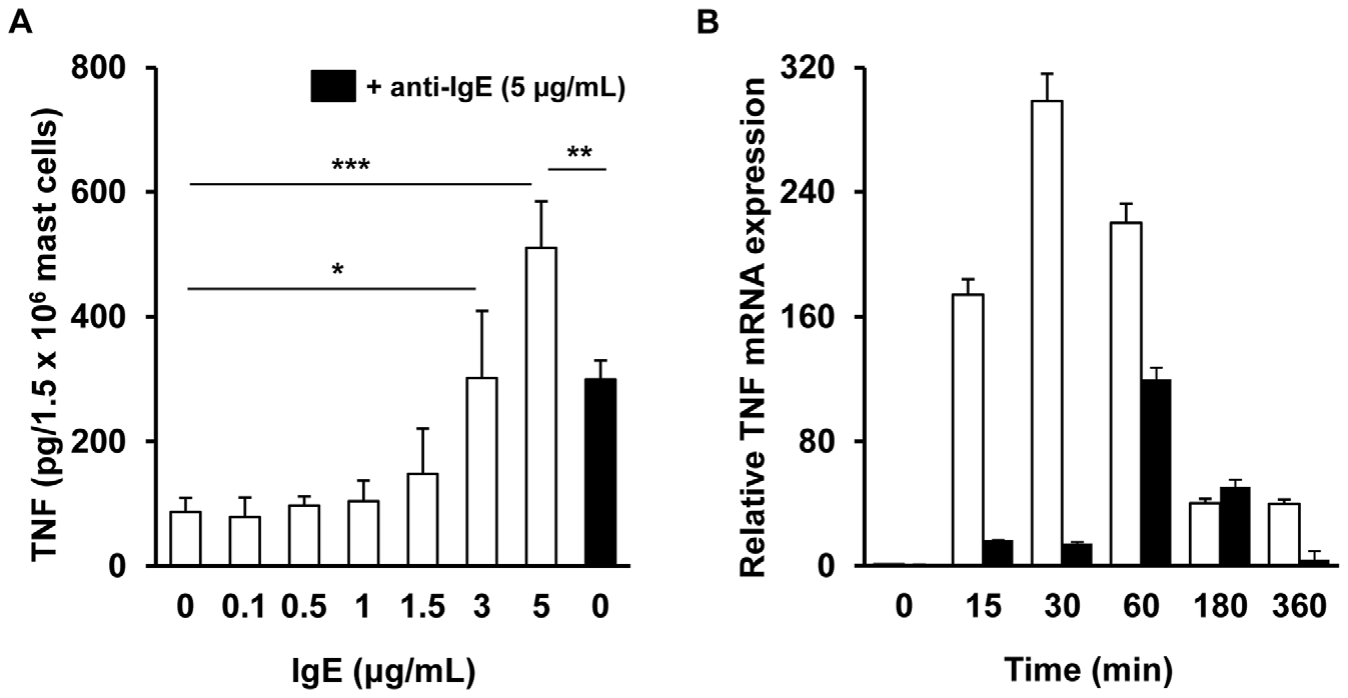
IgE alone induces *de novo* TNF synthesis in mast cells. Mast cells were incubated with different concentrations of IgE (white bars) for 6 h. Anti-IgE-induced TNF secretion (black bar) is shown as a positive control (A). For TNF mRNA assessment mast cells were incubated with IgE (white bars) or anti-IgE (black bars) both at 5 µg/mL for the indicated times (B). TNF mRNA levels were determined by qRT-PCR and presented as fold-increase over the value of cytokine mRNA expression in non-stimulated cells after normalization with the transcript level of the housekeeping gene rat Actb. Results are presented as the mean ± SEM of at least three separate experiments performed in duplicate (A) or triplicate (B) (n≥3). *p<0.05, **p<0.01, ***p<0.001.

Next, we investigated whether IgE alone, used at different concentrations, stimulates mast cells to generate and release newly-synthesized arachidonic acid metabolites and stimulates *de novo* TNF production. As shown in figure 2 IgE at lower concentrations (from 0.1 µg/mL to 3 µg/mL) did not activate cysLT generation in mast cells. IgE alone, used at a concentration of 5 µg/mL, stimulated significant cysLT synthesis in mast cells, comparable to anti-IgE-induced cysLT production. We also found that IgE alone at concentrations of 0.1, 0.5, 1, and 1.5 µg/mL was incapable of triggering significant TNF production by mast cells during 6 h incubation. In contrast, substantial amounts of TNF were secreted from mast cells treated with IgE at higher concentrations, i.e. 3 µg/mL and 5 µg/mL. Interestingly, level of TNF release in mast cells stimulated with IgE at 5 µg/mL was significantly (p<0.01) higher than that induced by anti-IgE (Fig. 3A). In order to confirm that IgE alone induces *de novo* TNF synthesis, the expression of TNF mRNA was analyzed. qRT-PCR was carried out and the relative expression of TNF mRNA in IgE-stimulated mast cells (5 µg/mL) compared to non-stimulated cells was assessed. It was revealed that IgE alone was capable of elevating TNF mRNA expression in mast cells in a time-dependent manner (Fig. 3B). TNF mRNA reached a peak level after 30 min of stimulation with IgE (up to 298.47±17.57-fold as compared to non-stimulated cells). Moreover, IgE alone was more potent at up-regulating TNF mRNA expression than anti-IgE-mediated TNF mRNA expression in mast cells at most examined incubation times.

### IgE Alone Influences Mast Cell Migration

**Figure 4:**
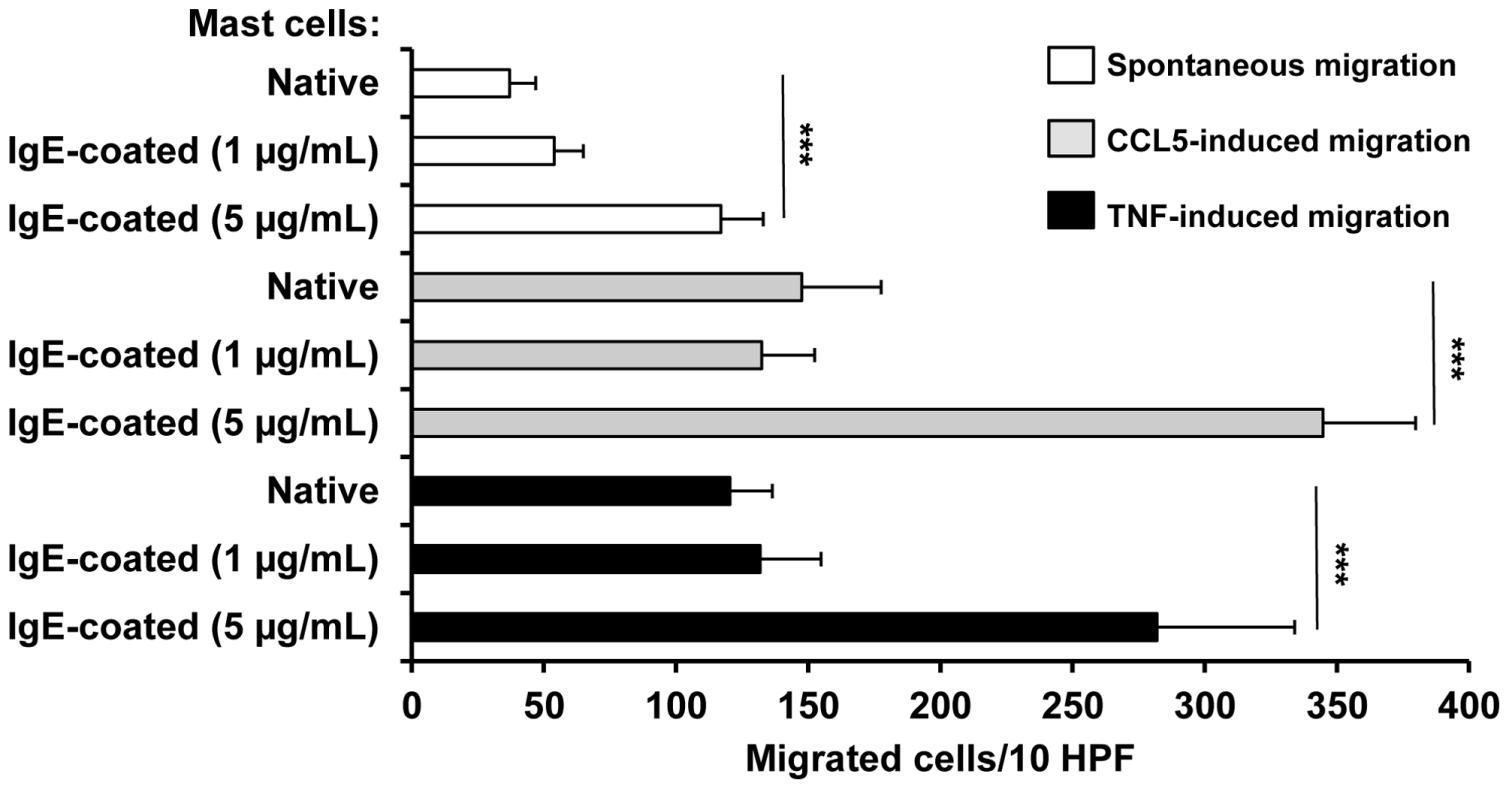
IgE alone affects mast cell migration. Mast cells were pretreated with IgE at 1 µg/mL or 5 µg/mL (IgE-coated mast cells) or medium (native mast cells) for 1 h before being placed into the upper wells, whereas medium (spontaneous migration), CCL5 or TNF had been added to lower wells of Boyden microchamber. Results are presented as the mean ± SEM of at least four independent experiments and each experiment was carried out in duplicate (n≥4). ***p<0.001.

We also conducted experiments to study the influence of IgE alone, used at 1 µg/mL and 5 µg/mL, on mast cell spontaneous migration as well as on CCL5- and TNF-induced mast cell migratory response. The results of these experiments are shown in figure 4. Only slight spontaneous migration of native mast cells was observed on laminin-coated filters. As expected, both CCL5, at a concentration of 100 ng/mL, and TNF, at a concentration of 10 ng/mL, induced significant migratory response of native mast cells. Mast cell treatment with 1 µg/mL IgE had no effect on spontaneous nor CCL5- and TNF-induced mast cell migration. We found, however, that pretreatment of mast cells with 5 µg/mL of IgE strongly enhanced both spontaneous and induced migration. Spontaneous migratory response of IgE-coated mast cells was approximately 3-times more potent than the native mast cell response, whereas CCL5- and TNF-dependent migration was 2.3-fold higher when cells were previously coated with IgE at 5 µg/mL, as compared to native mast cell migration (p<0.001) (Fig. 4).

### Cell Signaling Inhibitors Influence IgE-induced Mast Cell Activity

To gain some insight into the intracellular pathways through which IgE alone stimulates mast cells to degranulation, cysLT production, and TNF synthesis, as well as affects migratory response of mast cells, we examined the impact of an array of inhibitors specific to some selected signaling molecules. A number of inhibitors was employed: PD98059 and SB203580 - MAPK pathway inhibitors specific to ERK and p38 molecules, U73122 - an inhibitor of PLC/PLA_2_, LY294002 - an inhibitor of PI3K, and Src I-1 - a Src kinase inhibitor. Both Src I-1 and U73122 effectively reduced IgE-induced mast cell degranulation whereas PD98059 only partially attenuated histamine release induced by IgE alone, but to a statistically significant degree (p<0.05). ERK inhibitor and Src kinase inhibitor almost completely abolished, and PLC/PLA_2_ inhibitor significantly decreased, cysLT generation mediated by IgE alone. Mast cell pretreatment with Src I-1 or SB203580 resulted in a statistically significant (p<0.001) inhibition of IgE-induced TNF production and release. By contrast, ERK inhibitor was found to slightly attenuate IgE-mediated TNF synthesis (Table 1).

**Table 1:** Inhibition of IgE-induced mast cell histamine release, cysLT generation and TNF synthesis by various inhibitors.

	Inhibitor	Concentration (μM)	IgE (5 µg/mL)-induced mast cell activity	Inhibition (%)^1^
Histamine release (%)	None	0	13.8±3.0	-
	U73122	0.1	3.5±1.3	74.6**
	PD98059	100	7.5±2.4	45.7*
	Src I-1	0.01	3.8±1.0	72.5***
CysLT generation	None	0	518.8±75.4	-
(pg/1.5×10^6^ mast cells)	U73122	0.1	171.8±31.7	66.9**
	PD98059	100	67.5±32.1	87.0***
	Src I-1	0.01	63.5±28.4	87.8***
TNF synthesis	None	0	510.3±74.9	-
(pg/1.5×10^6^ mast cells)	SB203580	100	90.5±15.9	82.3***
	PD98059	100	427.5±7.1	16.2*
	Src I-1	0.01	75.3±14.4	85.2***

1 IgE-induced mast cell activity was referred to as 100%. *p<0.05, **p<0.01, ***p<0.001.

**Figure 5:**
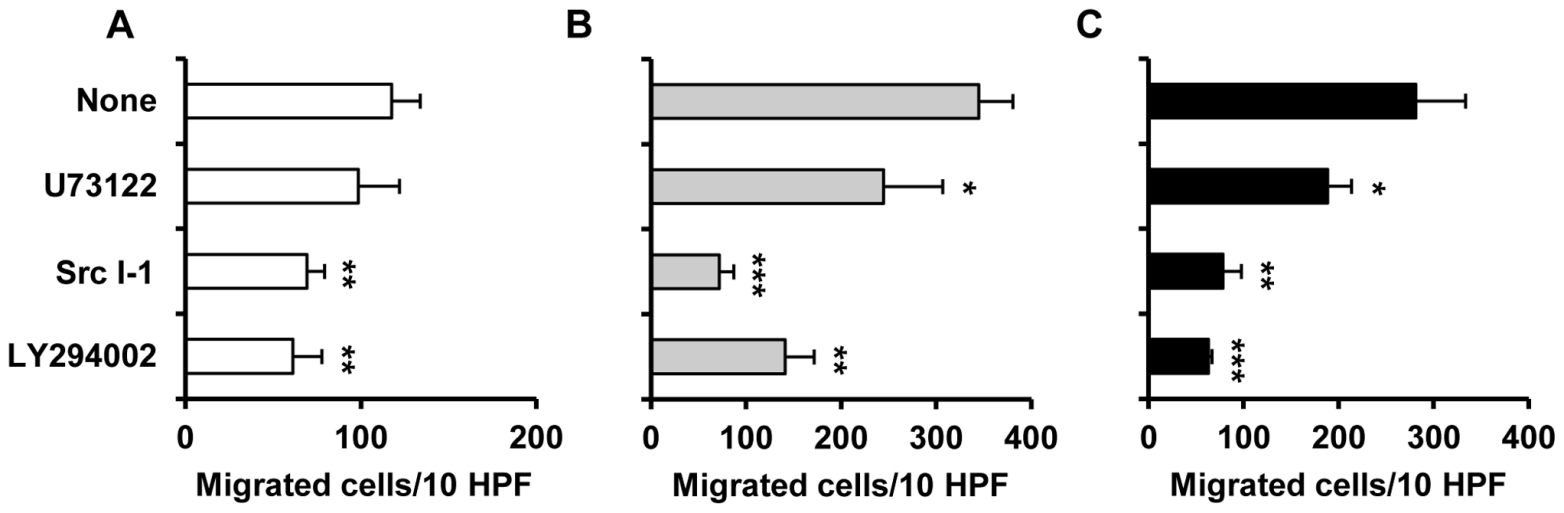
Cell signaling inhibitors influence IgE-affected both spontaneous and induced mast cell migration. Mast cells were preincubated with U73122 (0.1 µM), Src I-1 (0.01 µM), LY294002 (50 µM) or medium alone for 1 h (none). Then, mast cells were treated with IgE at 5 µg/mL, washed and spontaneous (A), CCL5-induced (B) and TNF-induced (C) migration was examined in a Boyden microchamber. Bars for the positive controls demonstrate the same data set as in figure 4. Results are presented as the mean ± SEM of four independent experiments and each experiment was carried out in duplicate (n = 4). *p<0.05, **p<0.01, ***p<0.001.

We also found that preincubation of mast cells with PLC/PLA_2_ inhibitor decreased CCL5- as well as TNF-induced migration of IgE-coated mast cells to a partial but statistically significant degree (p<0.01). The same inhibitor did not affect spontaneous mast cell migration. Src inhibitor and PI3K inhibitor both caused significant (p<0.01; p<0.001) reduction in CCL5-induced, TNF-induced as well as in spontaneous migration of IgE-coated mast cells (Fig. 5).

**Figure 6:**
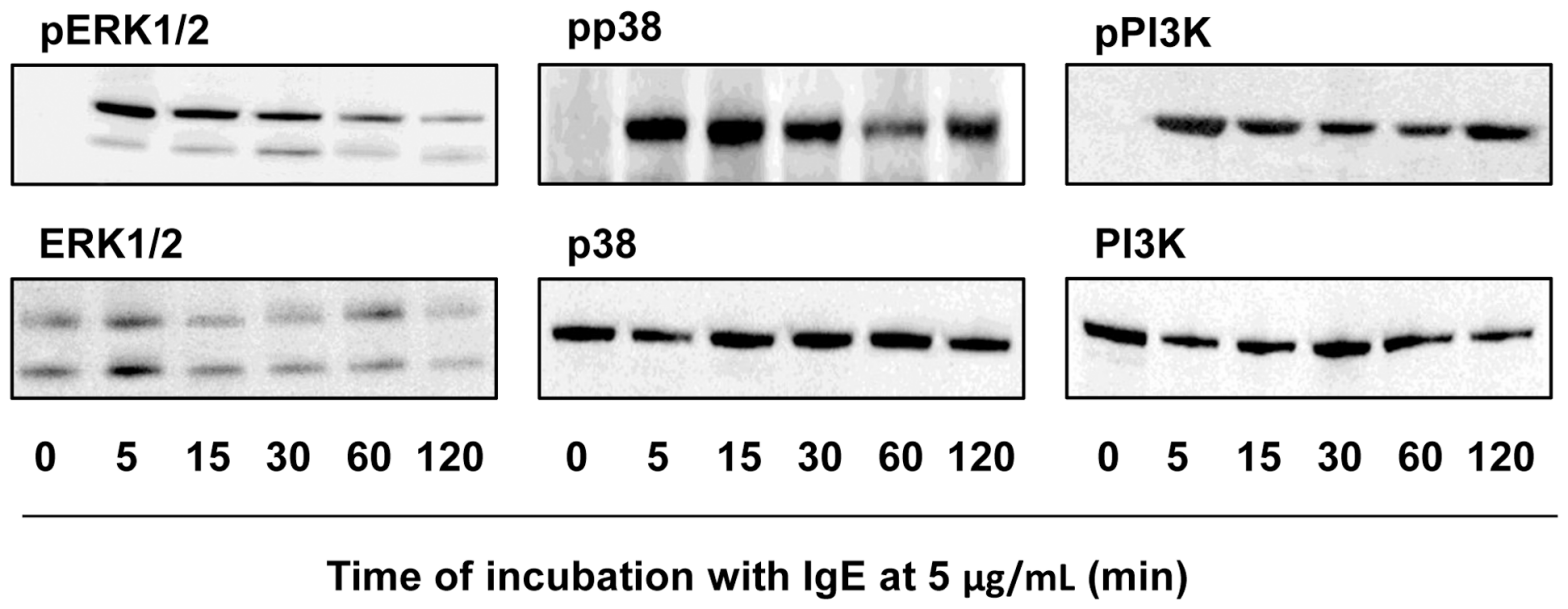
IgE alone stimulates phosphorylation of ERK, p38 and PI3K in mast cells. Mast cells were treated with IgE alone at 5 µg/mL for the indicated times. Total cell lysates (50 µg) were subjected to Western blotting analysis using anti-phospho antibodies specific to ERK1/2 (pERK1/2), p38 (pp38) or PI3K (pPI3K). The same blots were reprobed with respective antibodies that recognize these signaling molecules, irrespective of the phosphorylation states. Results are representative of 3 independent experiments.

Having established that IgE-mediated mast cell response is dependent on the activity of some signaling molecules, time-course experiments were performed to examine phosphorylation events of MAP kinases (ERK1/2, p38) and PI3K upon stimulation with IgE alone. As can be observed in figure 6, IgE at 5 µg/mL triggered the phosphorylation of all examined molecules within 5 min. Phosphorylation of p38 and PI3K remained at high levels at 120 min of treatment with IgE, whereas ERK1/2 were strongly phosphorylated in mast cells up to 60 min of stimulation.

### IgE Alone Modulates FcεRI Expression on Mast Cells

**Figure 7:**
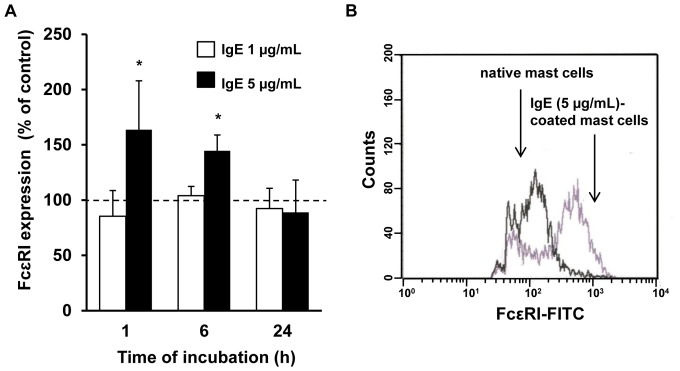
IgE alone up-regulates FcεRI expression on rat mast cells. Mast cells were incubated with IgE at 1 µg/mL (white bars) and 5 µg/mL (black bars) or without IgE (control FcεRI expression on native mast cells referred to as 100%) for 1, 6 and 24 h. Surface FcεRI expression was analyzed by flow cytometry using mouse anti-rat FcεRI primary antibodies (10 µg/mL) and FITC-labeled goat anti-mouse secondary antibodies (5 µg/mL) (A). Representative flow cytometry histogram showing FcεRI expression on native mast cells as well as on mast cells treated with 5 µg/mL for 1 h (B). Results are presented as the mean ± SEM of four independent experiments (n = 4). *p<0.05.

In the current study, the FcεRI expression was evaluated on native mast cells as well as on mast cells exposed to IgE used at concentrations of 1 µg/mL and 5 µg/mL for 1, 6 and 24 h. Flow cytometry analysis revealed that native mast cells expressed surface FcεRI receptor. We found that the baseline level of FcεRI expression was significantly up-regulated (p<0.05) upon 1 h incubation with IgE at 5 µg/mL, and reached 163.6±44.3% of control FcεRI expression on native mast cells. The treatment of mast cells with 5 µg/mL of IgE following a 6 h incubation resulted in a statistically significant (p<0.05) increase in FcεRI level, compared with the control unstimulated mast cells. Interestingly, the FcεRI expression on mast cells after prolonged exposure (24 h) to IgE at 5 µg/mL was comparable with control expression. The exposure of mast cells to 1 µg/mL of IgE did not cause alteration in surface FcεRI level, at any time point examined (Fig. 7A, Fig. 7B).

## Discussion

The central dogma of mast cell biology holds that FcεRI cross-linking by specific IgE and antigen initiates signaling cascade activation, leading not only to immediate preformed mediator secretion, but also to *de novo* synthesis and release of eicosanoids, cytokines and chemokines [2], [20]. Recent studies have indicated that once the FcεRI-IgE complex is formed, many alterations in mast cell activity occur. Firstly, it is well established that IgE causes enhancement of surface FcεRI expression on mast cells. More detailed studies have revealed that IgE-induced FcεRI up-regulation is the consequence of receptor stabilization and accumulation at the plasma membrane, which in turn leads to lower FcεRI internalization and degradation [22]–[25]. We demonstrated herein that IgE alone substantially increased constitutive expression of FcεRI on mature mast cells freshly isolated from rat peritoneal cavity.

In our study we also stated that IgE alone directly triggered mature rat mast cells to preformed mediator secretion, as assessed by histamine release, and cysLT production. Importantly, the release of mediators was substantial only when IgE at 5 µg/mL was employed. So far, only a handful of studies have examined the direct effect of IgE on mast cell degranulation and eicosanoid generation, and the results remain ambiguous. Some earlier studies have shown that IgE by itself induced BMMCs and human lung mast cells (HLMCs) degranulation, however only when mast cells were stimulated with at least 3 µg/mL of IgE [28], [33]. On the other hand, Kalesnikoff et al. [26] detected no considerable preformed mediator release from BMMCs, even if IgE was used at a concentration of 10 µg/mL. Although it was shown that mast cell stimulation with higher concentrations of IgE resulted in cysLT synthesis [28], [33], other studies have demonstrated no significant effect of IgE exposure on eicosanoid production in BMMCs [26]. Matsuda et al. [34] did not observe cysLT release from human umbilical CBMCs either, but it may be assumed that IgE concentration used was too low (2.5 µg/mL) to elicit mast cell response. There is strong evidence that IgE can induce the synthesis and release of some cytokines, including interleukin (IL)-2, IL-4, IL-6, IL-13, but not IL-5, and some chemokines, i.e. CCL2 and CXCL8 [26], [28], [32]–[34]. BMMC stimulation with IgE also resulted in significant *de novo* TNF synthesis and release [26], [28], [34]. Most importantly, Kalesnikoff et al. [26] stated that IgE was even more effective than FcεRI cross-linking with IgE and antigen at inducing TNF synthesis. Herein, we established that 3 µg/mL of IgE caused considerable production of TNF, and interestingly IgE at 5 µg/mL was able to induce far more potent *de novo* TNF generation than anti-IgE stimulation at both TNF mRNA and TNF released levels.

Under physiological conditions, the mast cell number in tissues is high and relatively constant [20], [21]. However, in some circumstances, mast cell accumulation is observed. Particularly, local mast cell hyperplasia is a prominent feature of allergic processes and the host response to parasite infections, i.e. conditions when IgE level is raised [2], [3]. Hence, our observations concerning the effect of IgE alone on mast cell migratory response are especially important. We have stated, for the first time ever, that IgE by itself directly altered mast cell motility when employed at higher concentration, i.e. 5 µg/mL. Spontaneous migratory response of mast cells coated with IgE increased by 216% compared with the native mast cell response. Similarly, CCL5- and TNF-induced migration of IgE-coated mast cells was strongly amplified and was approximately 2.3-fold higher than native mast cell induced migration. Previously, it has been shown that native mast cells are attracted to IgE [31] and mast cells sensitized with antigen- specific IgE migrate toward this antigen [31], [40]. In this paper we demonstrated that IgE by itself increased both undirected and directed mast cell migration. Thus, IgE molecules influence, in different ways, migration of mast cells, and thereby IgE can be implicated in local mast cell accumulation, especially in the course of allergic processes.

The precise signaling mechanisms underlying mast cell response mediated by IgE alone have yet to be clarified. It is highly likely, however, that IgE-dependent signaling events are similar, if not the same, to the classical pathway cascade elicited by FcεRI aggregation [41]. Firstly, FcεRI β and γ subunits, which are responsible for signal amplification and transduction from FcεRI [41], were found to be tyrosine-phosphorylated by IgE alone in BMMCs [26], [42]. What is more, Kohno et al. [27] demonstrated that molecules deeply involved in the initiation of FcεRI-mediated signals i.e. Src family kinases (Lyn and Syk), were essential for IgE-induced BMMC survival. Similarly, IgE-induced IL-6 production was significantly inhibited due to Syk-mediated signal depletion in BMMCs [28], [32]. In our study, we have provided evidence that Src kinases were crucial for the IgE-driven release of preformed mediators, cysLT as well as *de novo*-synthesized TNF. The Src kinase-initiated signal also appeared to be vital for IgE-affected rat peritoneal mast cell increased migration towards CCL5 and TNF. Interestingly, spontaneous migratory response of IgE-coated mast cells was only partially abrogated through the withdrawal of Src kinases signal.

It was established that IgE alone triggered the phosphorylation of PKB (also known as Akt) and several MAP family kinases, i.e. ERK, p38, JUN N-terminal kinase (JNK) [26], [28]. It also initiated the rise in cytosolic Ca^2+^ in a PLC-dependent fashion [32], [33], [35], [42]. In our study the phosphorylation of ERK1/2, p38 and PI3K was observed when mast cells were stimulated with IgE alone. On the other hand, Asai et al. [22] observed no phosphorylation of PKB or MAP kinases after BMMC treatment with IgE. Only a few papers have assessed which downstream signaling molecules are implicated in particular mast cell activities mediated by IgE alone. Inhibitors specific to PLC and PI3K were found to diminish IgE-mediated β-hexosaminidase release from BMMCs [42], and our findings revealed that IgE-induced degranulation of fully mature rat mast cells was heavily dependent on PLC, and in part on ERK. To our knowledge, there is no data regarding to the participation of specific molecules in IgE-dependent eicosanoid generation and release. We indicated that IgE-induced cysLT release required the activity of both PLC/PLA_2_ and ERK i.e. signaling molecules deeply involved in arachidonic acid metabolism [41]. The release of newly-synthesized IL-6 as a result of BMMC stimulation with IgE was significantly blocked after PI3K, PKC, ERK and p38 signal withdrawal [26], [32]. Our findings that both p38, and to a lesser extent ERK, were involved in IgE-induced *de novo* TNF generation are consistent with these observations. We also documented that IgE-affected spontaneous migratory response was independent of PLC/PLA_2_, and was only partially generated through the PI3K-involved pathway. Alternatively, the effect of IgE alone on the CCL5- and TNF-induced migration of mast cells was mediated by PI3K and to some extent by PLC/PLA_2_. These discrepancies in the mechanistic basis between spontaneous and induced migratory responses of IgE-coated mast cells may be a result of additional/common pathways triggered *via* chemoattractant receptors.

In conclusion, our results, taken in concert with previous data, clearly indicated that IgE by itself influences mast cell activity and releasability. It should be emphasized, however, that IgE alone effectively influences mast cell biology only when used at higher concentrations. Of note, there are some circumstances when IgE level is elevated *in vivo*, and so mast cell FcεRI occupation with IgE can be relatively high. Under these conditions, IgE *per se* may act as a mast cell activating factor. Moreover, since it is well-known that FcεRI is present not only on mast cells but also on basophils, eosinophils, neutrophils, monocytes, and on different subsets of dendritic cells [43], it may be assumed that IgE may regulate the response of other FcεRI-bearing cells. Indeed, recent studies have indicated that some cellular activities of basophils and dendritic cells are modulated by IgE molecules [44], [45]. Undoubtedly, the repertoire of biological effects elicited by IgE needs to be further clarified.
